# Preserved fine-tuning of face perception and memory: evidence from the own-race bias in high- and low-performing older adults

**DOI:** 10.3389/fnagi.2014.00060

**Published:** 2014-04-04

**Authors:** Jessica Komes, Stefan R. Schweinberger, Holger Wiese

**Affiliations:** DFG Research Unit Person Perception and Department of General Psychology and Cognitive Neuroscience, Friedrich Schiller University of JenaJena, Germany

**Keywords:** face perception, face memory, cognitive aging, N170, own-race bias, expertise

## Abstract

Previous research suggests specific deficits in face perception and memory in older adults, which could reflect a dedifferentiation in the context of a general broadening of cognitive architecture with advanced age. Such dedifferentiation could manifest in a less specialized face processing system. A promising tool to investigate the fine-tuning of face processing in older age is the own-race bias (ORB), a phenomenon reflecting more accurate memory for own-relative to other-race faces, which is related to an expertise-based specialization of early perceptual stages. To investigate whether poor face memory in older age is accompanied by reduced expertise-based specialization of face processing, we assessed event-related brain potential correlates of the ORB in high- vs. low-performing older adults (mean age = 69 years; *N* = 24 per group). Intriguingly, both older groups demonstrated an equivalent pattern of a behavioral ORB, and a parallel increase in N170 for other-race faces, reflecting less efficient early perceptual processing for this face category. Group differences only emerged independent of face ethnicity: whereas low-performers exhibited a right-lateralized N170, high-performers showed a more bilateral response. This finding may suggest a compensatory mechanism counteracting age-related decline in face perception enabling more efficient encoding into memory in high performers. Overall, our results demonstrate that even a less efficient face processing system in older adults can exhibit preserved expertise-related specialization toward own-race faces.

## Introduction

During aging, the human brain undergoes various changes in structure, function, and neural transmission (Sowell et al., 2003; Raz et al., 2005). These alterations most probably underlie, and certainly covary with cognitive functioning (Cabeza et al., 2004; Dennis and Cabeza, 2008; Nyberg et al., 2012). During childhood, the interaction of maturation, experience, and learning results in cortical differentiation (for review, see Johnson, 2001; Scherf et al., 2007), which is paralleled by more fine-grained cognitive abilities (Li et al., 2004; Werkle-Bergner et al., 2009). This pattern reverses during older age, when senescent changes include cognitive and sensorimotor dedifferentiation (Lindenberger and Baltes, 1994; Baltes and Lindenberger, 1997; Lindenberger et al., 2001) and diminished cortical specialization (Park et al., 2004, 2012).

Critically, age-related alterations and their putative consequences can be very diverse, and within-cohort differences can be similarly pronounced as between-cohort differences (Salthouse, 2013). More specifically, while some older adults show decreased performance in memory and executive processes (for overview, see Anderson and Craik, 2000), others maintain high levels of functioning, and match (see e.g., Cabeza et al., 2002; Duarte et al., 2006; Friedman et al., 2010) or even outperform their younger counterparts (Christensen et al., 1999; Henry et al., 2004; Federmeier et al., 2010). Results from functional brain imaging suggest that different processes in the aging brain could mediate such performance differences: First, reduced activations of memory-related brain regions were found in older adults with poor performance (for review, see Grady, 2008). Second, increased activations in older participants were observed in prefrontal areas contralateral to those usually activated in younger adults (for reviews, see Cabeza, 2002; Park and Reuter-Lorenz, 2009; Reuter-Lorenz and Park, 2010; but see Nyberg et al., 2010, for potentially discrepant results from longitudinal data). Importantly, these latter findings were interpreted as indexing either dedifferentiation or compensation (for reviews, see Reuter-Lorenz and Cappell, 2008; Park and Reuter-Lorenz, 2009). While dedifferentiation connotes “negative plasticity” in terms of loss in specialization of neuro-cognitive structures, compensation connotes “positive plasticity,” allowing additional resource recruitment to optimize performance (Reuter-Lorenz and Park, 2010). This debate remains largely unresolved (Davis et al., 2012).

In line with the dedifferentiation view, several studies on object and face processing suggested the fading of distinctive neural representations with increasing age in ventral visual cortex. Of note, activation in fusiform gyrus, which is category-sensitive for faces in younger adults, was less selective in older adults (Park et al., 2004, 2012; Chee et al., 2006). Other studies used event-related potentials (ERPs) to examine the face-sensitive N170, a negative occipito-temporal peak at approximately 170 ms after stimulus onset (Bentin et al., 1996; Eimer, 2011) to elucidate age-related changes in face processing. At potential variance with the above-described neuroimaging results, N170 category-selectivity (with larger N170 to faces than objects) was similarly found across age groups (Gao et al., 2009; Daniel and Bentin, 2010), suggesting preserved neural sensitivity for faces in older adults^1^. However, the N170 is sometimes increased in older adults (Gao et al., 2009; Daniel and Bentin, 2010; Wiese et al., 2013a; see also Rousselet et al., 2009, who reported larger amplitudes in older participants slightly after the N170 peak), and is also larger for inverted relative to upright faces, which is commonly interpreted as reflecting disrupted configural face processing. Notably, smaller N170 inversion effects were also observed in older participants (Gao et al., 2009; Daniel and Bentin, 2010). Hence, a larger N170 for upright faces and a smaller inversion effect in older adults may indicate a general reduction in early stages of face perception with higher age, potentially affecting subsequent higher-order cognitive functions, such as memory (see also Chaby et al., 2011). Consequently, the degree of preserved vs. deficient face perception in older adults might be reflected in differential face memory performance. To our knowledge, this idea has not been addressed in previous work.

The ability to distinguish between exemplars within a face category and to discriminate individual faces develops early, with a specialization toward own species faces from around 6 months of age (Pascalis et al., 2002) and subsequent further tuning toward own-race faces during childhood (see e.g., Scherf and Scott, 2012). In adults, the own-race bias (ORB) reflects the consistent finding of better memory for own-relative to other-race faces (for overview, see Meissner and Brigham, 2001). However, whether an ORB can still be observed in older adults has been scarcely investigated (Wallis et al., 2012). Age-related dedifferentiation could result in a less specialized face processing system (particularly in those older adults with poor face memory), and therefore in a reduced ORB, given that this effect is commonly interpreted to reflect expertise-related fine tuning of face processing mechanisms (Valentine and Endo, 1992; Tanaka et al., 2013). Here, we examined memory for own- and other-race faces in two groups of high- and low-performing older adults to test whether poor face memory in low-performers reflects advancing dedifferentiation (as indexed by a reduced or absent ORB). In addition, we addressed the question, whether reduced memory performance is associated with deteriorated perceptual processing as indexed by the N170.

Several recent studies have identified neural correlates of the ORB using ERPs, and N170 seems to play a prominent role in the generation of this effect. Suggestive of reduced early perceptual processing, other-race faces were found to elicit a larger N170 than own-race faces (Balas and Nelson, 2010; Stahl et al., 2010; Caharel et al., 2011). In a recent study, we reported a larger N170 to other-race faces during the learning phases of a recognition memory paradigm, in both young Asian and Caucasian participants (Wiese et al., 2014). Importantly, this effect during encoding significantly correlated with the subsequent recognition advantage for own-race faces during test. Apart from the ORB, an own-age bias (OAB) in terms of better memory for own-age relative to other-age faces has also been consistently described (for reviews, see Rhodes and Anastasi, 2012; Wiese et al., 2013b). To avoid underestimating older adults' performance, we decided to examine their face recognition memory in a full-factorial design by using young and old own- and other-race faces (Wiese, 2012).

The aim of our investigation was two-fold: First, we tested whether low- relative to high-performing participants would exhibit a reduced (or even absent) behavioral ORB which would argue for a (incipiently) dedifferentiated face processing system in this participant group. Second, we examined whether N170 effects would co-vary with memory performance, indicating less efficient early face perception in those older participants with lower memory performance.

## Materials and methods

### Participants

Forty-eight older participants (27 females, mean age = 69.0, *SD* = 4.7), recruited in senior citizen groups and via a press release in a local newspaper, participated in the study and were reimbursed with 7.50 Euro per hour. All participants were Caucasian and reported to reside in independent living conditions with little or no contact to Asian people. Participants were right handed according to a modified version of the Edinburgh Handedness Inventory (Oldfield, 1971). None reported psychiatric or neurological disorders or received central acting medication, and all participants reported normal or corrected-to-normal vision (visual acuity and contrast sensitivity were also formally tested, see below). Furthermore, all participants gave written informed consent and the study was approved by the local ethics committee.

The participant group was *post-hoc* subdivided via a median-split with respect to overall performance (mean *d*′ across experimental conditions) in the main recognition experiment into 24 high-performing (14 females, mean age = 68.4, *SD* = 5.2) and 24 low-performing (13 females, mean age = 69.6, *SD* = 4.4) older adults. The groups did not differ with respect to age, *F* < 1, or education, Mann–Whitney-*U* = 234.50, *p*_(masymptotic)_ = 0.240.

### Stimuli

Stimuli were identical to those used in Wiese (2012) and consisted of 480 gray-scale pictures showing 120 older Caucasian, 120 older eastern Asian, 120 young Caucasian, and 120 young eastern Asian faces (50% female, respectively), which were collected from diverse internet sources. Due to this stimulus selection procedure, the exact age of the persons depicted is unknown. However, stimuli have been rated for age by young participants in our previous study (rated age of young Caucasian faces = 28.61; *SD* = 0.42, young Asian faces = 29.28, *SD* = 0.41, older Caucasian faces = 66.16; *SD* = 0.57, older Asian faces = 72.29, *SD* = 0.94). All of the pictures displayed front views of neutral or moderately happy faces and were edited using Adobe Photoshop™ removing all information (clothing, background, etc.) apart from the face which was subsequently pasted in front of a black background. All stimuli were framed within an area of 170 × 216 pixels (6.0 × 7.6 cm), corresponding to a visual angle of 3.8° × 4.8° at a viewing distance of 90 cm.

### Procedure

Prior to the recognition memory experiment, visual acuity and contrast sensitivity were measured for each participant via a computer-based test (FrACT, Version 3.5.5; Bach, 1996) at 90 cm viewing distance. Participants were asked to indicate the positions of Landolt's C gaps presented in different sizes (test for visual acuity) and gray-scales (contrast sensitivity). Visual acuity was determined by the logarithm of the minimum angle of resolution (logMAR). Contrast sensitivity was measured by Michelson Contrast scores, which refer to the difference between highest and lowest luminance values divided by the sum of the two values.

The procedure of the main experiment was identical to Wiese (2012). Participants were seated in a dimly lit, electrically shielded, and sound-attenuated chamber (400A-CT_Special, Industrial Acoustics, Niederkrüchten, Germany) with their heads in a chin rest. Approximate distance between eyes and computer screen was 90 cm. Each experimental session began with a series of practice trials on different stimuli, which were excluded from data analysis. On each trial, a face stimulus was presented for various durations (depending on study vs. test phases, see below), preceded by a fixation cross for 500 ms and followed by a blank screen for 500 ms indicating the end of a trial.

The main experiment consisted of 12 blocks, each divided into a study and a test phase. In each study phase 10 young and 10 older faces, 50% Caucasian and 50% Asian, respectively, were presented for 5000 ms each. Half of the participants were asked to categorize the face on the screen as fast and correctly as possible according to age (elderly vs. young), whereas the other half was asked to categorize the face on the screen as fast and correctly as possible according to ethnicity (Asian vs. Caucasian). Furthermore, participants were instructed to memorize the faces. Between learning and test phases a fixed break of 30 s duration was inserted. During each test phase all of the 20 faces from the directly preceding study phase and 20 new faces (50% older, 50% Asian) were presented for 2000 ms each. Participants were instructed to indicate as fast as possible and without compromising accuracy whether the faces have been encountered in the preceding study phase. Between each block, participants were allowed a self-timed period of rest. During study and test, stimuli were presented in a randomized order, and key assignment and allocation of stimuli to learned and non-learned conditions were counterbalanced across participants. During study phases, mean reaction time (RT, correct responses only) and accuracy was analyzed. Data from the test phases were sorted into hits (correctly identified studied faces), misses (studied faces incorrectly classified as new), correct rejections (CR, new faces correctly classified as new), and false alarms (FA, new faces incorrectly classified as studied). Measures of sensitivity (*d*′) and response bias (C) were calculated (Green and Swets, 1966).

### ERP recording and analysis

Thirty-two-channel EEG was recorded using a BioSemi Active II system (BioSemi, Amsterdam, Netherlands). The active sintered Ag/Ag-Cl-electrodes were mounted in an elastic cap. EEG was recorded continuously from Fz, Cz, Pz, Iz, FP1, FP2, F3, F4, C3, C4, P3, P4, O1, O2, F7, F8, T7, T8, P7, P8, F9, F10, FT9, FT10, TP9, TP10, P9, P10, PO9, PO10, I1, I2, with a 512-Hz sample rate from DC to 155 Hz. Please note that BioSemi systems work with a “zero-Ref” set-up with ground and reference electrodes replaced by a CMS/DRL circuit (for further information, see www.biosemi.com/faq/cms&drl.htm).

Contributions of blink artifacts were corrected using the algorithm implemented in BESA 5.1 (MEGIS Software GmbH, Graefelfing, Germany). EEG was segmented from −200 until 1000 ms relative to stimulus onset, with the first 200 ms as baseline. Trials contaminated by non-ocular artifacts and saccades were rejected from further analysis. Artifact rejection was carried out using the BESA 5.1 tool, with an amplitude threshold of 100 μV, as well as a gradient criterion of 75 μV. Remaining trials were recalculated to average reference, digitally low-pass filtered at 40 Hz (12 db/oct, zero phase shift), and averaged according to the following four experimental conditions during learning for the first six study blocks (see below): young Asian, young Caucasian, elderly Asian, elderly Caucasian. The mean number of trials contributing to an individual averaged ERP for these conditions was 27, 27, 26, and 27, respectively^2^. The minimum number of trials contributing to an individual waveform was 16.

In the resulting waveforms, mean amplitudes for P1 were determined at O1/O2 and between 110 and 160 ms, for N170 between 180 and 220 ms, and for a subsequent time window from 220 to 260 ms at TP9/P9/PO9 and TP10/P10/PO10. Extensive analyses were also performed throughout the entire epoch, which can be found in the Supplementary Material. Statistical analyses were performed by calculating mixed-model analyses of variance (ANOVA), with degrees of freedom corrected according to the Greenhouse-Geisser procedure where appropriate.

## Results

### Visual acuity/contrast vision

An ANOVA on the logMAR measure of visual acuity with the between-subjects factor group (high- vs. low-performers) revealed no significant difference, *F*_(1, 46)_ = 1.73, *p* = 0.195, η^2^_*p*_ = 0.04. Similarly, an ANOVA on the Michelson Contrast indicated no group difference, *F* < 1.

### Performance

Given the length of the experiment (>70 min plus preparation times for EEG) and frequent reports of exhaustion toward the end of the session, we conducted an initial ANOVA on *d*′ with the factor “block” (1–6 vs. 7–12) to test for potential effects of fatigue. A significant block effect, *F* = 6.85, *p* = 0.012; η^2^_*p*_ = 0.13, indicated decreased performance in the second half of the experiment. As overall performance was a critical aspect, we decided to analyze data in the first six blocks only, to avoid contamination of any effects by fatigue.

**Figure 1:**
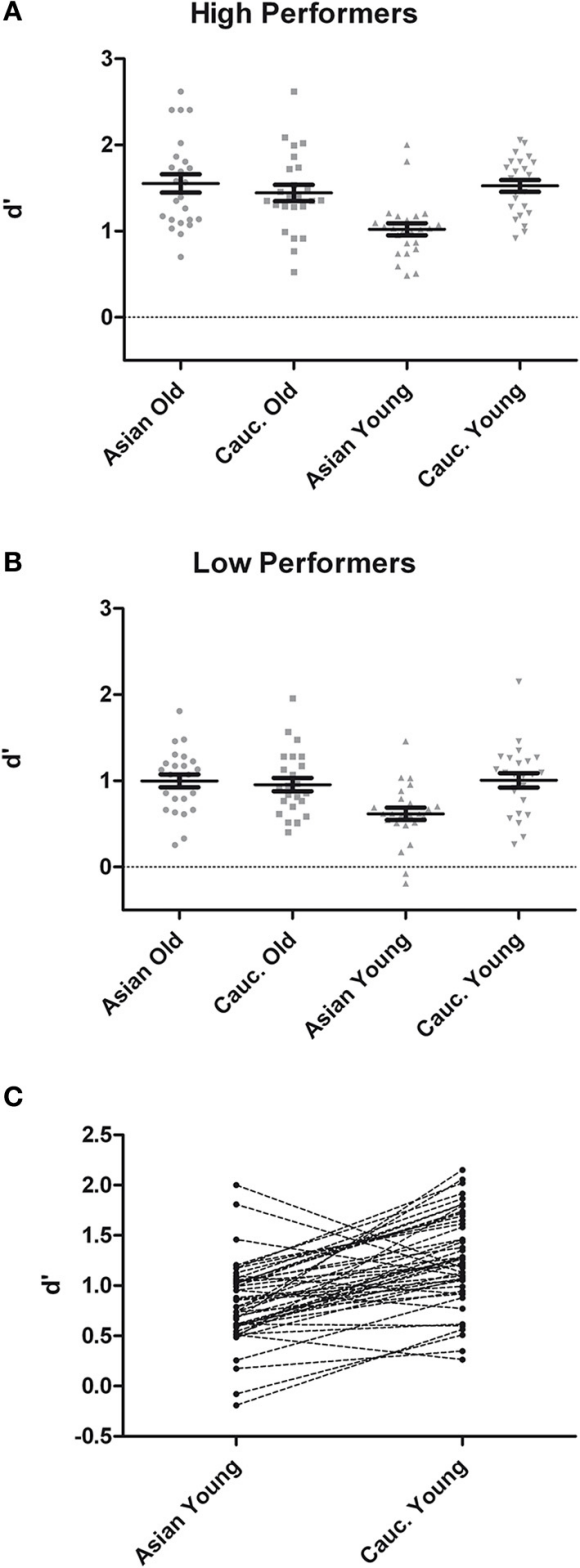
**Recognition memory performance measured in *d*′ for high- (A) and low-performers (B) during test phases from the recognition memory experiment**. **(C)** Pairwise differences in memory performance for young Asian vs. young Caucasian faces.

Analysis of *d*′ at test revealed main effects of group, *F*_(1, 46)_ = 55.42, *p* < 0.001, η^2^_*p*_ = 0.55, face ethnicity, *F*_(1, 46)_ = 11.06, *p* = 0.002, η^2^_*p*_ = 0.19, face age, *F*_(1, 46)_ = 11.62, *p* = 0.001, η^2^_*p*_ = 0.20, and a significant interaction of Face Ethnicity × Face Age, *F*_(1, 46)_ = 19.85, *p* < 0.001, η^2^_*p*_ = 0.30. *Post-hoc* tests indicated better recognition for young Caucasian vs. young Asian faces, *t*_(48)_ = 6.86, *p* < 0.001, *d* = 1.05, but no difference between older Caucasian and Asian faces, *t*_(48)_ = 1.18, *p* = 0.245, *d* = 0.20. Furthermore, recognition was more accurate for older Asian as compared to young Asian faces, *t*_(48)_ = 5.66, *p* < 0.001, *d* = 0.98, whereas there was no difference between older Caucasian and young Caucasian faces, *t*_(48)_ = 1.20, *p* = 0.237, *d* = 0.19. Importantly, these effects were not modulated by group, *F* < 1 (see Figure 1). As the division of participants into high and low performing groups may have led to a loss of statistical power due to dichotomization of the continuous memory score (see e.g., Cohen, 1983), we additionally calculated a correlation between overall *d*′ and the ORB for young faces, which revealed no significant result, *r* = 0.07, *p* = 0.62.

For the sake of completeness, we also analyzed response bias (C) but consider the finding as Supplementary Material.

### Event-related potentials

We analyzed the study phase ERPs, as previous research suggested the study phase N170 as a neural correlate of the ORB (Wiese et al., 2014). Note that effects involving topographic factors (hemisphere, site) are only reported when interacting with experimental factors. In accordance with our behavioral data analysis strategy, we restricted our ERP analyses to the first six blocks of the experiment.

A mixed-model ANOVA on P1 with the within-subject factors hemisphere (left, right), face ethnicity, and face age, and the between-subjects factor group resulted in no significant effects, *F* ≤ 3.62, *p* ≥ 0.064, η^2^_*p*_ ≤ 0.07.

**Figure 2:**
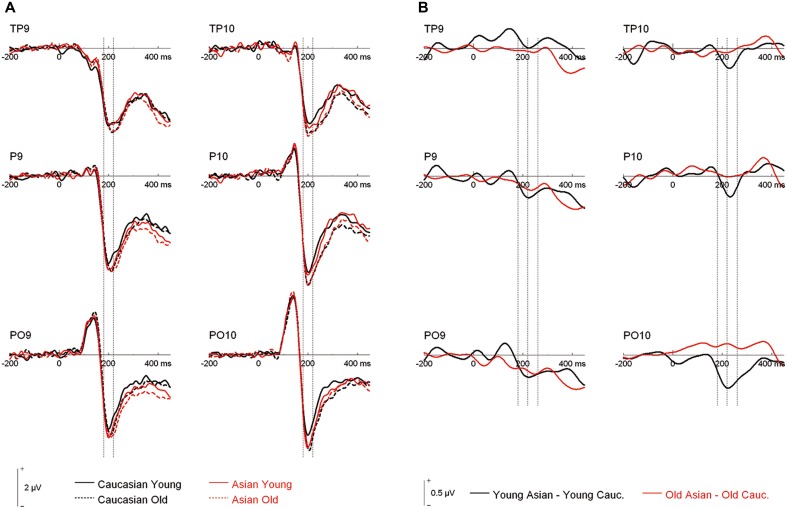
**(A)** Grand mean waveforms collapsed across all participants for young and older Asian and Caucasian faces from the learning phases of the recognition memory experiment. Dashed lines depict the 180–220 ms (N170) time window. **(B)** Difference curves depicting ERP ethnicity effects. Dashed lines depict the N170 (180–220 ms) and a directly following time window (220–260 ms).

**Figure 3:**
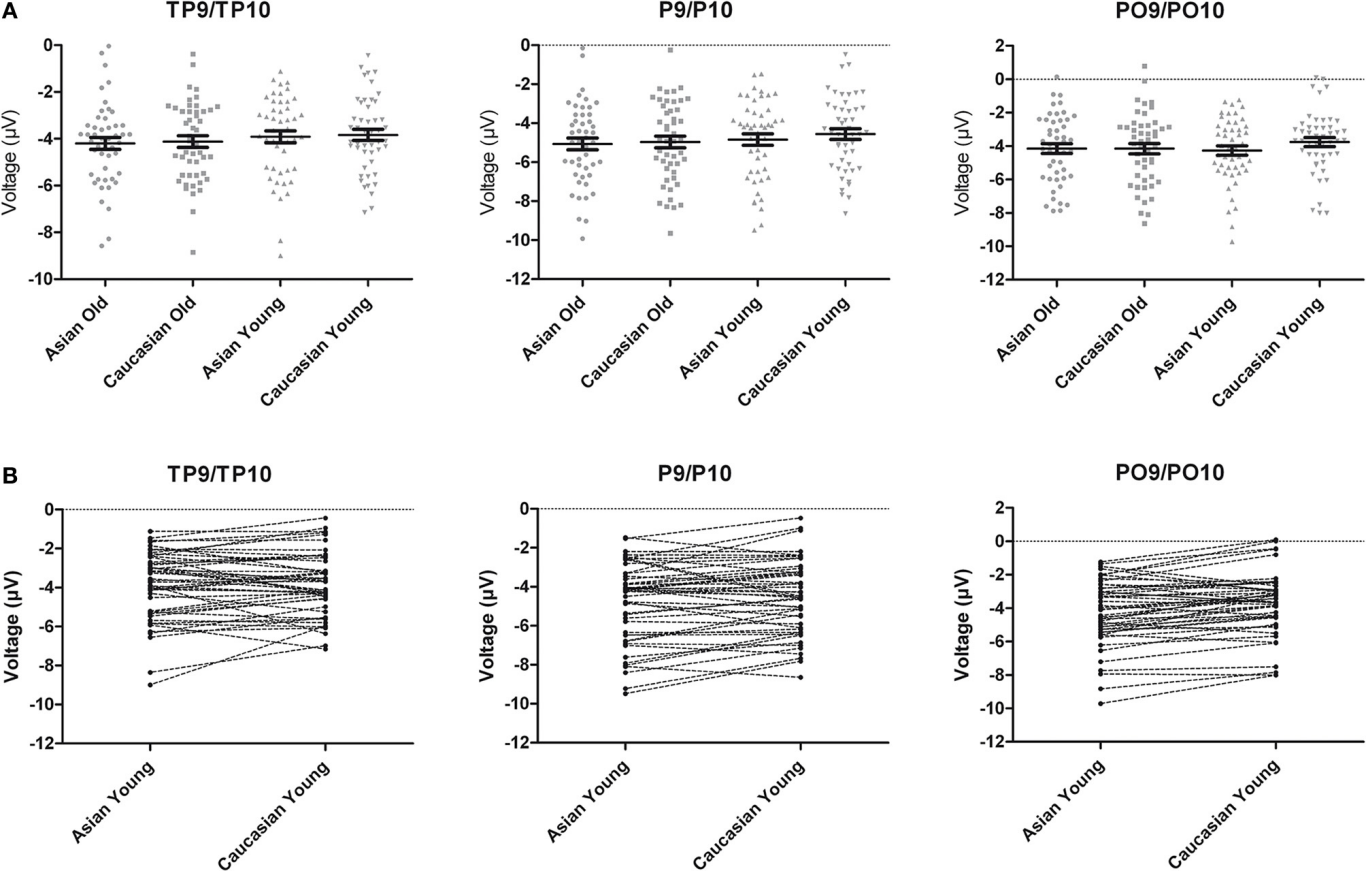
**(A)** Scatter plots depicting N170 amplitude averaged across left and right hemispheric occipito-temporal sites. Horizontal lines reflect mean N170 amplitudes, error bars denote standard errors of the mean. **(B)** Pairwise differences of N170 amplitude averaged across left and right hemispheric occipito-temporal sites for young Asian vs. young Caucasian faces.

An ANOVA on N170 with the within-subject factors hemisphere, site (TP/P/PO), face ethnicity and face age, and the between-subjects factor group resulted in effects of face ethnicity, *F*_(1, 46)_ = 5.77, *p* = 0.020, η^2^_*p*_ = 0.11, with larger N170 for Asian faces, and face age, *F*_(1, 46)_ = 8.88, *p* = 0.005, η^2^_*p*_ = 0.16, with larger N170 for old faces, as well as in an interaction of Face ethnicity × Face age × Site, *F*_(1.59, 73.01)_= 3.61 *p* = 0.042, η^2^_*p*_ = 0.073. Separate *post-hoc* analyses for each site and ethnicity yielded larger amplitudes for young Asian as compared to young Caucasian faces at P9/P10, *F*_(1, 47)_ = 4.46, *p* = 0.040, η^2^_*p*_ = 0.09 and PO9/PO10, *F*_(1, 47)_ = 15.45, *p* < 0.001, η^2^_*p*_ = 0.25, but not at TP9/TP10, *F* < 1. No corresponding effects were detected for older faces, all *F* < 1 (see Figures 2, 3).

**Figure 4:**
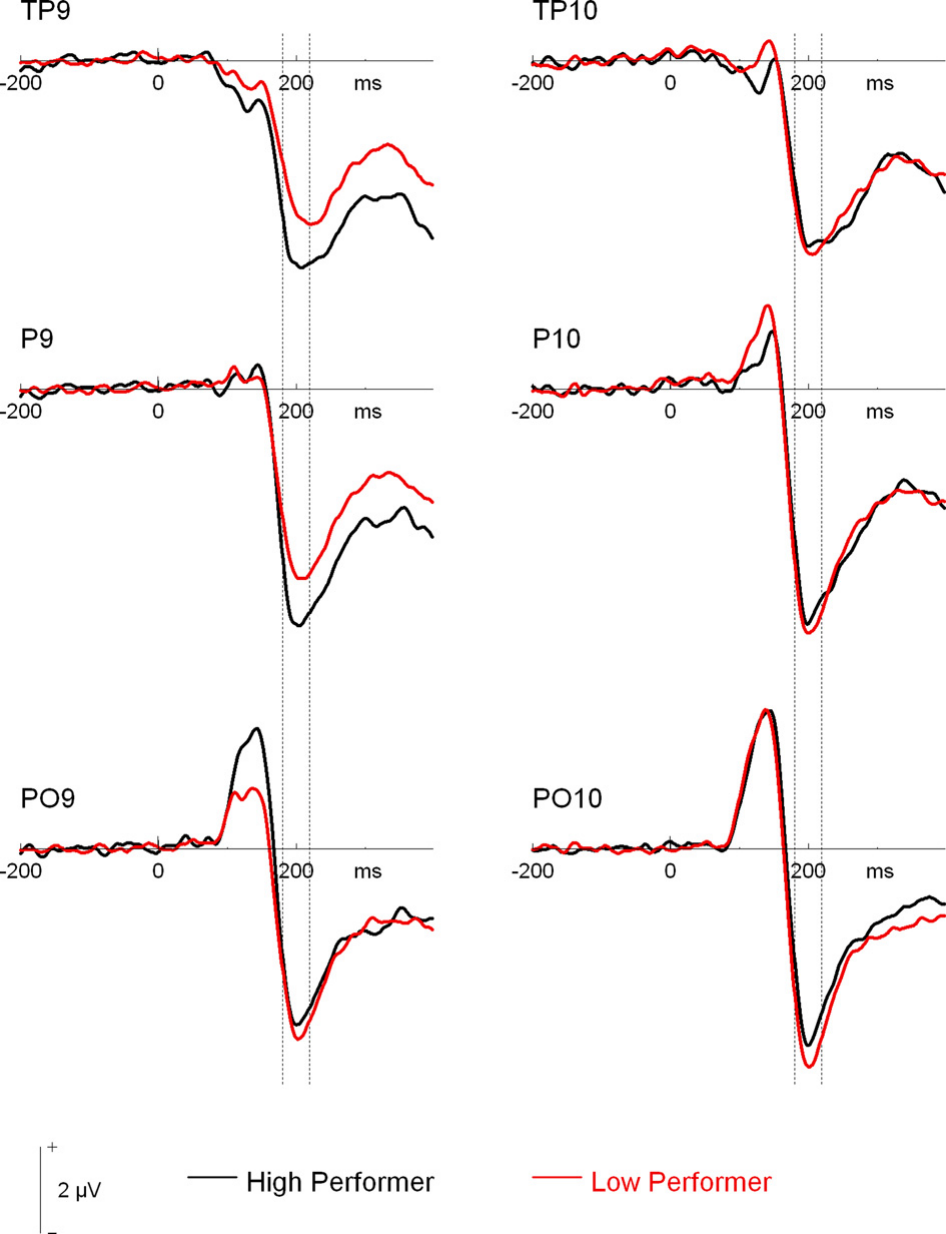
**Grand mean waveforms of high- and low-performing participants from the learning phases of the recognition memory experiment**. Dashed lines depict the 180–220 ms (N170) time window.

**Figure 5:**
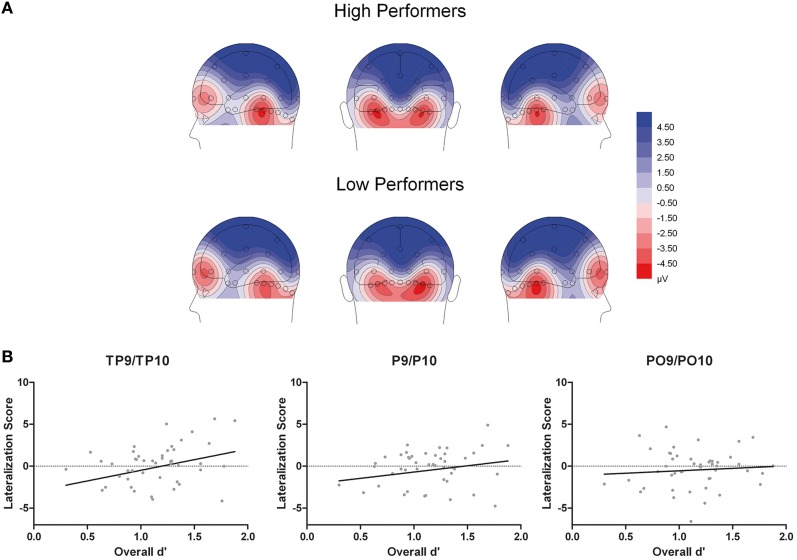
**(A)** Scalp topographical voltage maps (spherical spline interpolation, 110° equidistant projection) depicting N170 amplitude averaged across experimental conditions for high and low performing older adults. **(B)** Scatter plots depicting the correlations between N170 amplitude (right hemispheric - left hemispheric amplitudes across experimental conditions) and overall memory performance.

Additionally, the three-way interaction of Site × Hemisphere × Group, *F*_(1.44, 66.12)_ = 3.56, *p* = 0.048, η^2^_*p*_ = 0.072, was significant (see Figures 4, 5). Separate ANOVAs at each site and for high- and low-performers were suggestive of a right-lateralized N170 in the low-performers at TP9/TP10, *F*_(1, 23)_ = 3.27, *p* = 0.084, η^2^_*p*_ = 0.13, and at P9/P10, *F*_(1, 23)_ = 2.86, *p* = 0.104, η^2^_*p*_ = 0.11. By contrast, there was no evidence of right lateralization in high-performers, *F*_(1, 23)_ = 2.05, *p* = 0.166, η^2^_*p*_ = 0.08, who even exhibited numerically larger N170 over the left hemisphere at the more anterior electrode sites (e.g., for TP9/TP10; see Figure 4).

An additional analysis on N170 amplitude was carried out for the second half of the experiment to test whether group differences in N170 lateralization would hold with increasing fatigue. While the general pattern was similar to the first half (with larger left hemispheric amplitudes at more anterior sites for high relative to low performers), the respective mixed-model ANOVA revealed no significant interaction of Hemisphere × Site × Group, *F*_(1.47, 67.43)_ = 2.19, *p* = 0.134, η^2^_*p*_ = 0.045.

Moreover, given the continuous nature of the memory scores, we performed additional correlation analyses to establish a potential relationship between memory performance and ERP effects. First, to test whether the N170 ethnicity effect for young faces was related to overall performance, we calculated difference scores for N170 amplitude (Young Asian – Young Caucasian) at those electrode sites with significant effects in ANOVA reported above (P and PO). This measure was not correlated with overall performance, all *r*_(46)_ < 0.23, all *p* > 0.14, confirming the results from the median split analysis. Second, N170 lateralization scores (right hemispheric – left hemispheric amplitudes across experimental conditions) were calculated for TP, P, and PO sites separately. This measure correlated significantly with overall memory scores at TP, *r*_(46)_ = 0.29, *p* = 0.046^3^, but not at P, *r*_(46)_ = 0.20, *p* = 0.179, and PO sites, *r*_(46)_ = 0.08, *p* = 0.586 (see Figure 5B), again confirming the results from the median split analysis.

**Figure 6:**
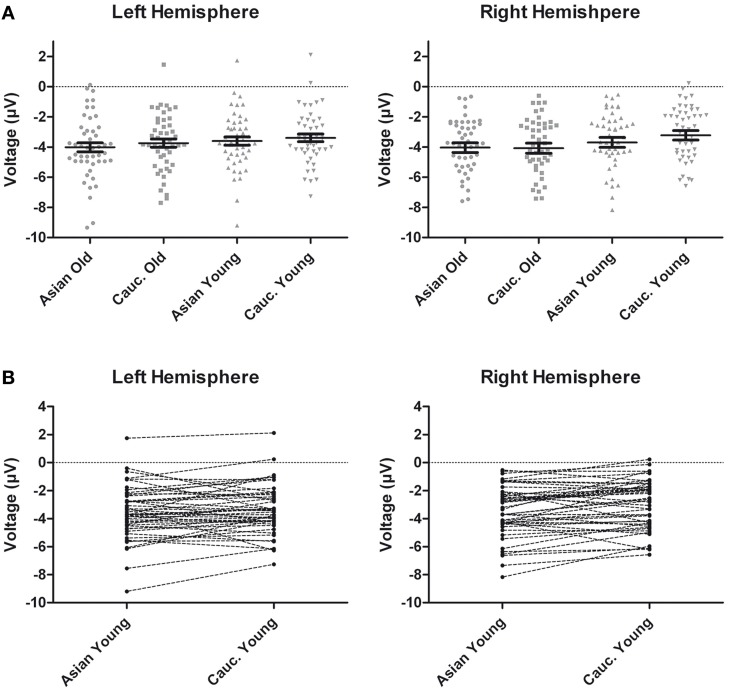
**(A)** Scatter plots depicting ERP amplitudes in the 220–260 ms time window averaged across left and right hemispheric occipito-temporal sites. Horizontal lines reflect mean amplitudes, error bars denote standard errors of the mean. **(B)** Pairwise differences of ERP amplitudes in the 220–260 ms time window averaged across left and right hemispheric occipito-temporal sites for young Asian vs. young Caucasian faces.

Following inspection of the ERP difference curves for the ethnicity effects (see Figure 2B), we performed an additional analysis using a time window from 220 to 260 ms. This analysis revealed main effects of face age, *F*_(1, 46)_ = 44.74, *p* < 0.001, η^2^_*p*_ = 0.49, and ethnicity, *F*_(1, 46)_ = 8.01, *p* = 0.007, η^2^_*p*_ = 0.15, which were qualified by a significant interaction of hemisphere × age × ethnicity, *F*_(1, 46)_ = 4.74, *p* = 0.035, η^2^_*p*_ = 0.09. Follow-up tests revealed significantly more negative amplitudes for young Asian relative to young Caucasian faces over the right, *F*_(1, 47)_ = 9.79, *p* = 0.003, η^2^_*p*_ = 0.17, but not over the left hemisphere, *F*_(1, 47)_ = 1.65, *p* = 0.206, η^2^_*p*_ = 0.03 (see Figure 6). No significant ethnicity effects were detected for old faces, neither over the left, *F*_(1, 47)_ = 3.35, *p* = 0.074, η^2^_*p*_ = 0.07, nor over the right hemisphere, *F* < 1. No significant effects involving the group factor were detected, all *F* < 2.03, all *p* > 0.15. Moreover, further analyses revealed no significant correlations between the lateralization scores and overall *d*′, TP9/TP10: *r* = 0.27, *p* = 0.062, P9/P10: *r* = 0.23, *p* = 0.12, PO9/PO10: *r* = 0.11, *p* = 0.47. Similarly, the ethnicity effect for young faces over the right hemisphere did not correlate with overall *d*′, *r* = −0.06, *p* = 0.68, supporting the above finding of no relation between ERP ethnicity effects and overall memory performance.

We also conducted extensive data analyses on the later P2 (260–400 ms), N2 (400–600 ms), and a late slow-wave (600–1000 ms) at occipito-temporal electrode sites. These analyses essentially replicated previous findings, with a prominent left lateralized ethnicity effect starting in the P2-time range, and a right lateralized age effect starting in the N2-time range (see Wiese, 2012). As these findings were not of primary interest for the present study, we refrain from a detailed report in the main paper (please see Supplementary Material).

## Discussion

The present study examined the ORB in face recognition memory in high- and low-performing older adults. Despite prominent overall performance differences, both groups demonstrated an equivalent magnitude of the ORB. Paralleling these behavioral results, ERP effects of face ethnicity first showed up in a larger N170 to young Asian vs. Caucasian faces in both groups alike. Additionally, performance-related ERP differences were observed between groups: While low-performing older adults showed a tendency for a right-lateralization of N170, this was not the case in high-performers. These findings are discussed in more detail below.

Our first aim was to test whether poor overall performance would be accompanied by dedifferentiation of processing within the category of faces, in terms of a reduced ORB in low-performers. Such a finding would complement previous reports of attenuated between-category (faces vs. objects) neural distinctiveness in older adults (Park et al., 2004, 2012), by indicating a degree of loss in the fine-tuning of the face processing system. We observed a clear ORB for young faces, which was virtually identical in both high- and low-performing participants. We therefore conclude that the fine-tuning of the face processing system of low-performers was not compromised when compared to high-performers, despite their reduced overall face memory. This conclusion is also in line with our finding of similar ethnicity effects in the N170 and the directly following time window in the two older groups (convergent with the additional result of no significant correlation of the ERP ethnicity effects with overall memory performance in both time segments), which further suggests similar encoding of facial race in the older groups.

Our observation of a preserved fine-tuning of the face processing system in older adults is generally in line with findings from an individual difference perspective. Hildebrandt et al. (2011) observed no dedifferentiation of individual differences in the latent constructs of face cognition as compared to general cognitive functioning. This finding was further substantiated in a subsequent study comparing object and face processing, in which only a slight dedifferentiation was observed, which was not specific for faces but operated on a more general cognitive level (Hildebrandt et al., 2013). In the present study, the behavioral ORB was equivalent between groups, although overall memory was reduced in low performers. Hence, we suggest that low performers did not exhibit poor face memory due to less specialized face processing, but rather due to more general cognitive decline.

Of note, although a previous study from our group found a significant correlation between the N170 ethnicity effect and the behavioral ORB (Wiese et al., 2014), two studies using a latent variable approach reported no relationship between the amplitude of the N170 and face cognition (Herzmann et al., 2010; Kaltwasser et al., in press). Yet, the latent variable approach produces combined measures of a number of tests, which may well tap into different processes than those reflected by N170 amplitude. More precisely, N170 is typically thought to represent very early perceptual processing stages (such as structural encoding or the detection of a face-like pattern, see e.g., Amihai et al., 2011; Eimer, 2011). Thus, less efficient processing of other-race faces at this early processing stage appears to result in a cascade of less efficient subsequent processes, which may ultimately result in less accurate recognition memory. Importantly, none of the tests used in the studies by Herzmann et al. (2010) or Kaltwasser et al. (in press) examined the processing of other-race faces. Early perceptual processing stages may have been highly efficient in all tasks used by Herzmann and others (Herzmann et al., 2010; Kaltwasser et al., in press), but not in the other-race conditions of the present and our previous study.

At a more specific level it is notable that ethnicity effects were prominent for young but not old faces, both in the N170 component and the subsequent time window, as well as in the behavioral ORB. The absence of an ORB for old faces in memory replicates two recent studies (Wallis et al., 2012; Wiese, 2012). We attribute this consistent absence of an ORB for old faces to decreased perceptual salience of ethnicity information in these faces, along with an increased salience of general age-related changes in facial shape and skin texture. The second aim was to examine whether the previously observed larger N170 amplitude in older as compared to young adults (e.g., Wiese et al., 2013a) was related to a decrease in early perceptual processing, which would in turn result in reduced face memory in older adults. This idea was based on the N170 inversion effect, reflecting an increased N170 amplitude for inverted, less configurally processed faces (e.g., Jacques et al., 2007), an effect which is reduced in older participants (Gao et al., 2009; Daniel and Bentin, 2010; Saavedra et al., 2012). In the present study, a decrease in face memory was not associated with larger N170 amplitudes, arguing against the idea of less efficient early face perception as the basis for reduced memory. If anything, N170 was larger in high- relative to low-performing participants, which, however, was apparent only over the left hemisphere.

Related to this latter finding, we detected a significant interaction involving the factors hemisphere and group, which suggested a more right-lateralized N170 in low-performers and a more bilateral pattern in high-performers. This finding was additionally confirmed in a correlation analysis, in which a more bilateral N170 was associated with higher overall performance. N170 is typically lateralized to the right hemisphere in young participants (e.g., Bentin et al., 1996; Amihai et al., 2011; Eimer, 2011), whereas a reduced lateralization of N170 amplitude in older adults has been previously described (Gao et al., 2009; Daniel and Bentin, 2010). Based on previous neuroimaging results (e.g., Cabeza, 2002; Cabeza et al., 2002) this finding has been interpreted as reflecting compensation of age-related decrements in face perception (Gao et al., 2009; Daniel and Bentin, 2010). The present study adds to this idea, by revealing that a reduced laterality of N170 can indeed relate to performance levels. Our finding is generally in line with the idea of reduced hemispheric asymmetry as a function of compensation rather than dedifferentiation (for reviews, see Cabeza, 2002; Reuter-Lorenz and Cappell, 2008). This may not only occur for higher-order cognitive processes, but also for (early) perceptual processes (De Sanctis et al., 2008) and the present findings extend this idea to the domain of face processing. However, although a similar pattern compared to the first half was observed, the interaction of hemisphere by group was no longer significant in the second half of the experiment, in which overall performance was clearly reduced in both groups. This finding may suggest that compensatory neural activity requires effortful processing and that fatigue hampers such activity in high performing older adults.

Although the more bilateral N170 in high-performers argues for a compensatory mechanism, the specific interpretation of their enhanced left-hemispheric N170 is subject to debate. One possibility considers that the left hemisphere may be more involved in feature-based than configural or holistic face processing (Rossion et al., 2000; Scott and Nelson, 2006). Accordingly, high-performers may engage in more feature-based processing during encoding, enabling them to exhibit better memory performance than low-performers at test. While such a strategy could provide more effective encoding fostering clearer subsequent representations, it is noteworthy that even high-performers in the present study perform at levels clearly below those of young participants in an identical experiment (see Wiese, 2012). Accordingly, even high-performers may not be able to fully compensate for age-related decline in face memory.

The present study is, to our knowledge, the first to examine ERP correlates of the ORB in older adult participants. The finding of an ORB independent of overall performance indicates that the fine-tuning of the face processing system toward faces of particular expertise is preserved in older adults. In line with this interpretation, the specific pattern of N170 ethnicity effects was found to parallel the behavioral ORB. In addition, a more bilateral N170 response in high-performing older adults suggests a partial compensation for general age-related decline in face perception by recruiting additional neural resources in the left hemisphere. In conclusion, the present results indicate that older adults' face processing system, even when working less efficiently, may still exhibit preserved expertise-related specialization toward own-race faces.

### Conflict of interest statement

The authors declare that the research was conducted in the absence of any commercial or financial relationships that could be construed as a potential conflict of interest.

## Footnotes

^1^Please note, that the above cited results obtained from fMRI and ERPs might not necessarily be discrepant, but may be reconciled, given that the N170 (unlike the N250 or N250r, see e.g., Schweinberger et al., 2004; Kaufmann et al., 2009) is most probably not generated in the fusiform gyrus. Please also note that these studies used peak measures to determine differences between age groups, which has been criticized (for alternative approaches, see Rousselet et al., 2009, 2011).

^2^Please note that only trials from the first half of the experiment were analyzed, see Results section.

^3^Please note that this was also significant when calculating a Spearman correlation, Rho = 0.29, *p* = 0.048, which is less sensitive to outliers as it uses ranked data.
