# Supplementary material for: Preserved fine-tuning of face perception and memory: evidence from the own-race bias in high- and low-performing older adults
Source: Front Aging Neurosci. 2014 Apr 4;6:60. doi: 10.3389/fnagi.2014.00060 (PMC3983485; doi:10.3389/fnagi.2014.00060)
Supplement: Supplementary file 1 [file DataSheet1.DOC]

**Supplementary Material**

**Results**

**Response Bias C**. A corresponding ANOVA on C revealed an interaction of Group x Face Ethnicity, *F*(1,46) = 4.95, *p =* .031, *η²*_p_ = .10 (see Table S1). Post-hoc tests yielded an effect of race with more conservative responses for Caucasian as compared to Asian faces in the high performing group *F*(1,23) = 5.04, *p =* .035, *η²*_p_ = .18, but not in the low-performing group, *F* < 1. In addition ethnicity interacted with age, *F*(1,46) = 48.63, *p <* .001, *η²*_p_ = .52. Follow-up testing indicated that responses were more conservative for young Asian as compared to young Caucasian faces, *t*(47) = 2.83, *p* = .007, *d* = 2.58, for older Caucasian as compared to older Asian faces, *t*(47) = 4.70, *p* < .001, *d* = 2.88, for young Asian as compared to older Asian faces, *t*(47) = 5.90, *p* < .001, *d* = 0.63 and for older Caucasian as compared to younger Caucasian faces, *t(*47) = -2.58 , *p =* .020, *d* = 0.33*.*

Table S1. Response Bias (C); Mean (SD).

|  | Caucasian Faces | Asian Faces |
| --- | --- | --- |
| Young Faces | -.25 (.37) | -.09 (.42) |
| Older Faces | .14 (.35) | - .12 (.40) |

**P2 (260 -400 ms).** A corresponding mixed-model ANOVA resulted in a main effect of face age, *F*(1, 46) = 14.84, *p* < .001, *η²*_p_ = .24, indicating more positive amplitudes for young as compared to old faces. Interestingly, an interaction of Hemisphere x Ethnicity, *F*(1, 46) = 8.02, *p* = .007, *η²*_p_ = .15, was detected. Further testing via ANOVAs for the left and right hemisphere separately revealed more positive amplitudes for Caucasian as compared to Asian faces at left hemispheric sites, *F*(1, 46) = 7.07, *p* = .011, *η²*_p_ = .13. No corresponding effect was found over the right hemisphere, *F*(1, 46) = 1.06, *p =* .308, *η²*_p_ = .02 (see Figure 2 in main paper).

**N2 (400-600 ms).** A corresponding ANOVA detected main effects of ethnicity, *F*(1, 46) = 11.58, *p* = .001, *η²*_p_ = .20, and age, *F*(1, 46) = 7.62, *p* = .008, *η²*_p_ = .14, which were further qualified by several interactions. First, a three-way interaction of Face Ethnicity x Face Age x Hemisphere, *F*(1, 46) = 9.64, *p=*.003, *η²*_p_ = .17, was observed, and follow-up testing again revealed more positive amplitudes for Caucasian as compared to Asian faces over the left, *F*(1, 46) = 19.88, *p* < .001, *η²*_p_ = .30 but not over the right hemisphere, *F* <1. Conversely, young faces elicited less negative amplitudes as compared to old faces over the right hemisphere only, *F*(1, 46) = 13.19, *p* = .001, *η²*_p_ = .22, whereas no such effect was detected over left hemispheric electrode sites, *F* <1 (see Figure S1a).

Second, an interaction of Site x Ethnicity x Group was found, *F*(1.74,79.98) = 3.64, *p* = .036, *η²*_p_ = .07. Follow-up ANOVAs conducted at the three electrode positions, and for high- and low-performer separately, indicated an effect of ethnicity at TP-electrodes in the low-performing group, *F*(1, 23) = 13.19, *p* = .001, *η²*_p_ = .22, with more negative amplitudes for Asian as compared to Caucasian faces. No such effect was apparent in the high-performing group, *F* < 1. At P-electrodes, Asian elicited more negative amplitudes than Caucasian faces in both low-, *F*(1, 23) = 10.43, *p* = .004, *η²*_p_ = .31, and high-performing groups, *F*(1, 23) = 4.62, *p* = .042, *η²*_p_ = .17. Similarly, at PO-electrodes Asian faces elicited more negative amplitudes as compared to Caucasian faces in both the low- , *F*(1, 23) = 5.95, *p* = .023, *η²*_p_ = .206 and high-performing group, *F*(1, 23) = 6.15, *p* = .021, *η²*_p_ = .21. Additionally, at TP- sites a trend for an effect of group, *F*(1, 46) = 3.06, *p* = .087, *η²*_p_ = .06, pointed towards more negative amplitudes for Asian faces in the low-performing relative to the high-performing group. Corresponding effects were neither detected for Asian faces at P- or PO- electrode sites, nor for Caucasian faces at any electrode position, all *F* < 1.

**(Positive) Slow-Wave (600-1000 ms).** A corresponding ANOVA revealed a main effect of age, *F*(1, 46) = 6.52, *p* = .014, *η²*_p_ = .12, pointing towards more negative amplitudes for old as compared to young faces. Interestingly, age interacted with hemisphere, *F*(1, 46) = 6.97, *p* = .011, *η²*_p_ = .13. Follow-up ANOVAs revealed that old faces elicited more negative amplitudes than young faces over the right hemisphere *F*(1, 47) = 16.08, *p* < .001, *η²*_p_ = .26, while no such effect was apparent over the left hemisphere, *F* < 1. Additionally, ethnicity interacted with hemisphere, *F*(1, 46) = 8.95, *p* = .004, *η²*_p_ = .16. Subsidiary ANOVAs revealed more negative waveforms for Asian as compared to Caucasian faces over the left, *F*(1, 47) = 8.06, *p* = .007, *η²*_p_ = .15, but not over the right hemisphere, *F*(1, 47) = 1.37, *p* = .248, *η²*_p_ = .23 (see Figure S1a).

The analysis also resulted in an interaction of Site x Ethnicity, *F*(1, 46) = 4.26, *p* = .017, *η²*_p_ = .09. Post-hoc tests showed that Asian faces elicited clearly more negative amplitudes as compared to Caucasian faces at PO9/PO10, *F*(1, 47) = 4.61, *p* = .037, *η²*_p_ = .09, while only a trend for a corresponding ethnicity effect was observed at electrodes P9/P10, *F*(1, 47) = 3.22, *p* = .079, *η²*_p_ = .06. No ethnicity effect was apparent at TP9/TP10, *F* < 1.

Finally, there was an interaction of Ethnicity x Age x Group, *F*(1, 46) = 4.77, *p* = .034, *η²*_p_ = .09. ANOVAs carried out for the high- and low- performing groups and for young and old faces separately revealed more positive amplitudes for old Caucasian faces as compared to old to Asian faces, *F*(1, 23) = 11.17, *p* = .003, *η²*_p_ = .32, in the high-, but not in the low-performers, *F* < 1. No such effect was apparent for young faces neither in the high-performing nor in the low-performing group, both *F* < 1. Trends for effects of performance group pointed towards more negative amplitudes in the low-performing relative to the high-performing group for older Caucasian faces, *F*(1, 46) = 3.49, *p* = .068, *η²*_p_ = .07, for young Asian faces, *F*(1, 46) = 2.87, *p* = .097, *η²*_p_ = .06), for young Caucasian faces, *F*(1, 46) = 2.92, *p* = .094, *η²*_p_ = .06, but not for older Asian faces, *F* < 1 (see Figure S1b and c).

Figure S1a.

**
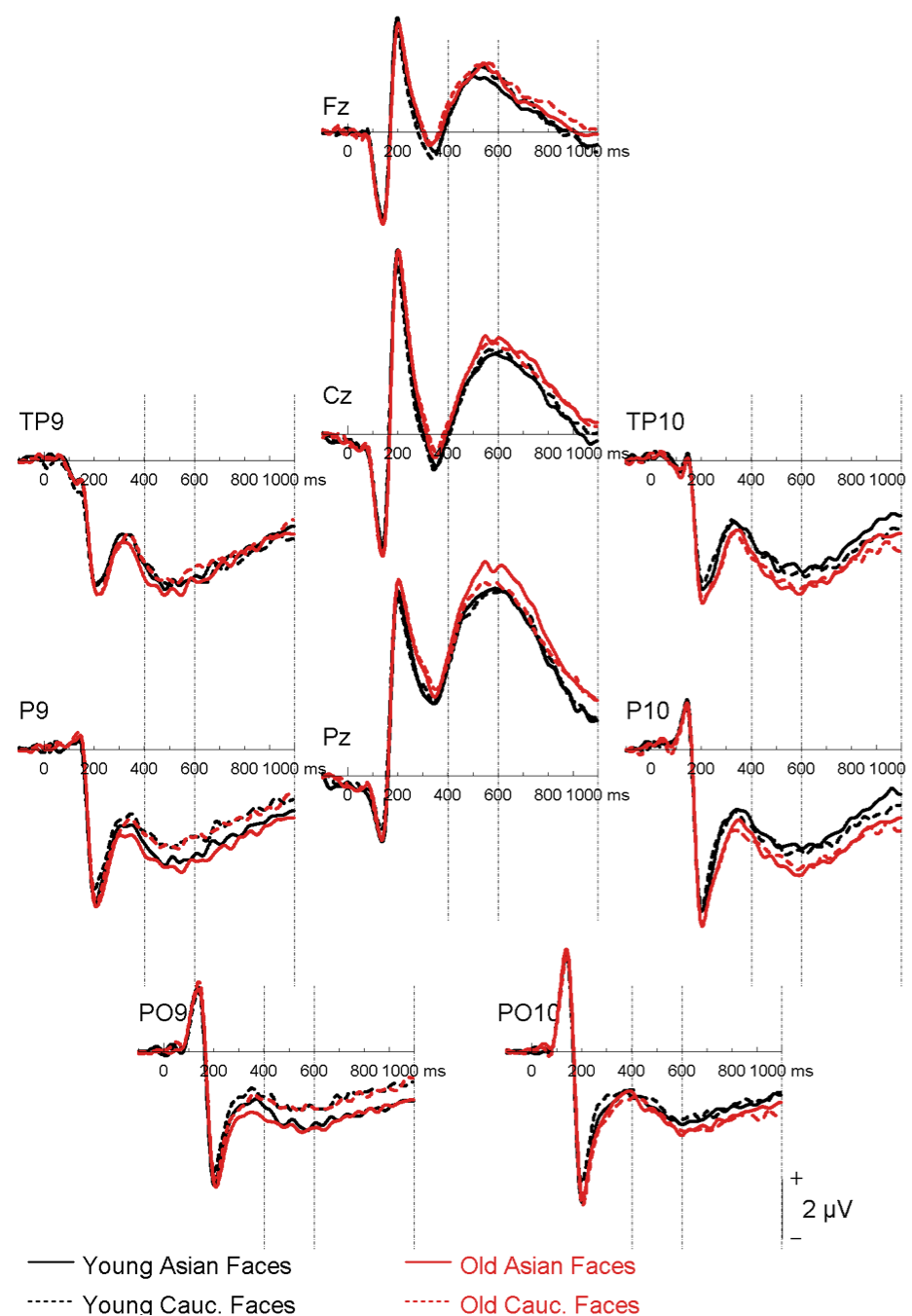
**

Figure S1b.

**
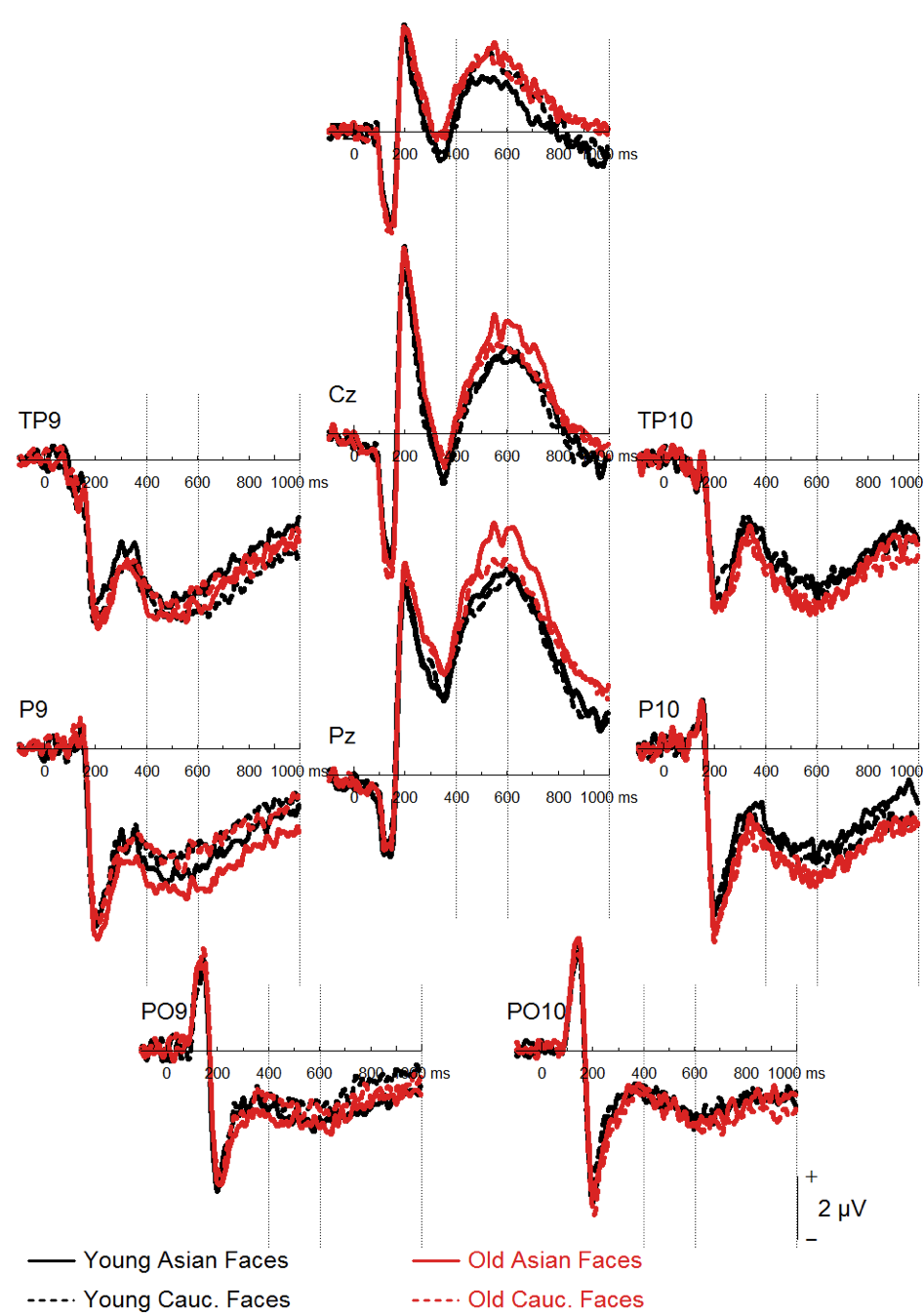
**

Figure S1c.


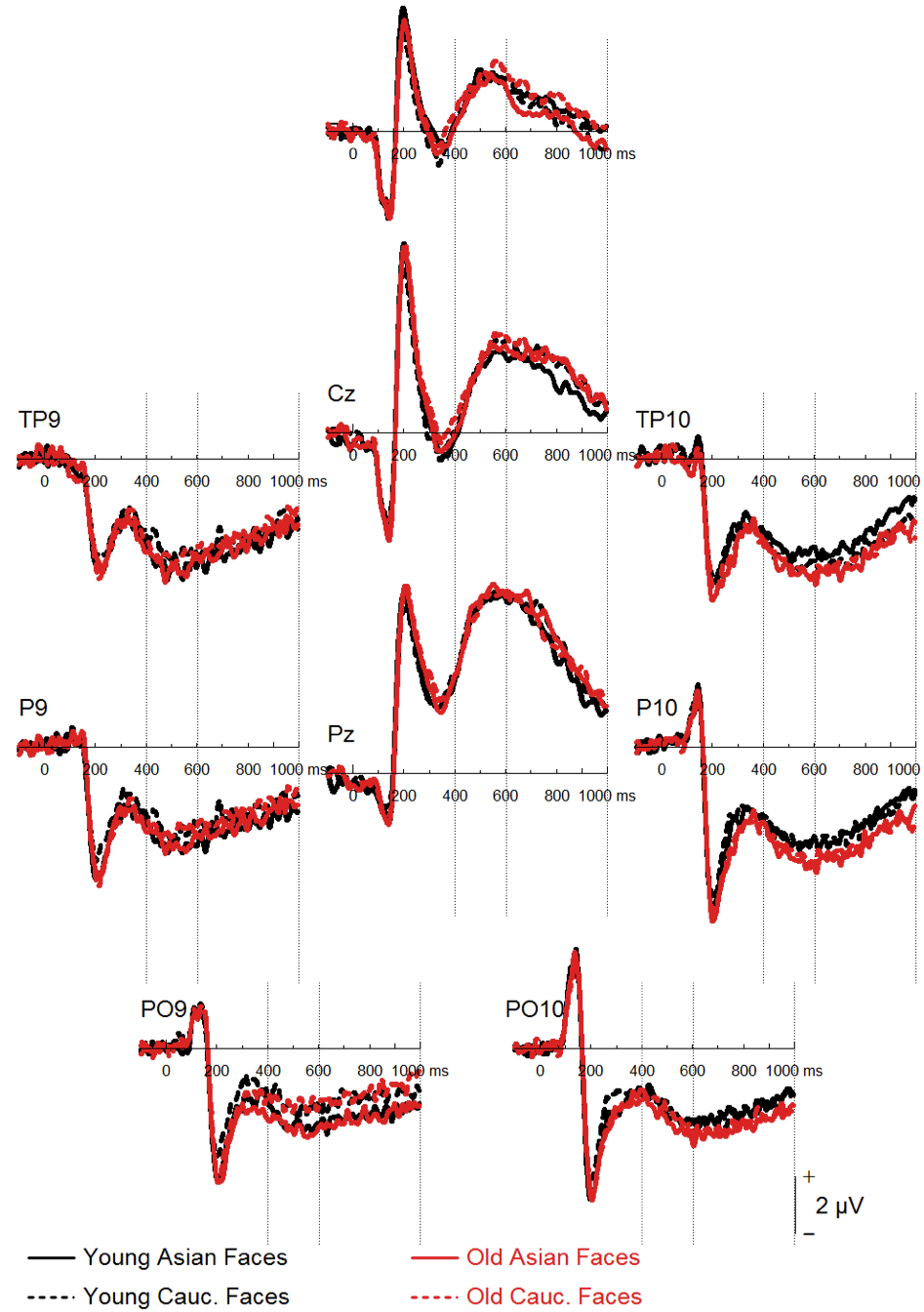


**Supplementary Figure Captions**

Supplementary Figure 1a (Figure S1a). Grand Mean waveforms collapsed across older participants for young and older Asian and Caucasian from the learning phases of the recognition memory experiment. Dashed lines depict the 400-600 ms (N2), and 600-1000 ms (positive slow-wave) time window.

Supplementary Figure 1b (Figure S1b). Grand Mean waveforms of high-performing participants for young and older Asian and Caucasian from the learning phases of the recognition memory experiment. Dashed lines depict the 400-600 ms (N2), and 600-1000 ms (positive slow-wave) time window

Supplementary Figure 1c (Figure S1c). Grand Mean waveforms of low-performing participants for young and older Asian and Caucasian from the learning phases of the recognition memory experiment. Dashed lines depict the 400-600 ms (N2), and 600-1000 ms (positive slow-wave) time window.
